# The Current and Future Treatment of Brain Metastases

**DOI:** 10.3389/fsurg.2016.00030

**Published:** 2016-05-25

**Authors:** Douglas A. Hardesty, Peter Nakaji

**Affiliations:** ^1^Department of Neurosurgery, Barrow Neurological Institute, St. Joseph’s Hospital and Medical Center, Phoenix, AZ, USA

**Keywords:** brain metastasis, chemotherapy, personalized medicine, radiotherapy

## Abstract

Brain metastases are the most common intracranial malignancy, accounting for significant morbidity and mortality in oncology patients. The current treatment paradigm for brain metastasis depends on the patient’s overall health status, the primary tumor pathology, and the number and location of brain lesions. Herein, we review the modern management options for these tumors, including surgical resection, radiotherapy, and chemotherapy. Recent operative advances, such as fluorescence, confocal microscopy, and brachytherapy, are highlighted. With an increased understanding of the pathophysiology of brain metastasis come increased future therapeutic options. Therapy targeted to specific tumor molecular pathways, such as those involved in blood–brain barrier transgression, cell–cell adhesion, and angiogenesis, are also reviewed. A personalized plan for each patient, based on molecular characterizations of the tumor that are used to better target radiotherapy and chemotherapy, is undoubtedly the future of brain metastasis treatment.

## Introduction

Nearly 200,000 patients are newly diagnosed with brain metastases annually in the United States, and metastases of the lung, skin, kidney, breast, and gastrointestinal tract are the most common intracranial malignancies (1, 2). Historically, overall survival after diagnosis is poor; however, in the last 30 years, improved systemic disease therapies and multimodality brain metastasis treatment have substantially increased survival. This increase in the *quantity* of life after diagnosis allows clinicians to minimize morbidity and focus on the patient’s *quality* of life. Choosing an appropriate personalized treatment plan for patients with brain metastasis maximizes survival and minimizes morbidity from unnecessary or futile treatments. The wide variety of tumor types, treatment strategies, and constant innovations within the field requires close collaboration among neurosurgeons, medical oncologists, radiation oncologists, and other specialists. Current treatment paradigms for brain metastases employ several treatment modalities, including open surgical resection, Gamma Knife or CyberKnife stereotactic radiosurgery, focused external beam radiotherapy, whole-brain radiotherapy (WBRT), traditional chemotherapy, and newer targeted biological agents personalized for tumor type. We review the current standards of care for brain metastases and summarize modern advances in their intraoperative diagnosis and treatment (Table 1). Lastly, we provide an overview of recent basic science and translational research leading to better understanding of the personalized biology of brain metastasis through modern genomic, transcriptomic, and proteomic techniques.

**Table 1:** **Modern challenges in the multimodality management of brain metastasis**.

Challenge in brain metastasis management	Potential consequence to patient	Modern treatment solution(s)
Identification of microscopic brain–tumor interface at time of surgery	Residual micrometastasis left behind despite resection	Intraoperative fluorescence, handheld confocal microscopy
Inability to target tumor bed with external beam radiation due to radiosensitive structures, prior radiation, etc.	Out-of-field recurrence, or conversely, radiation toxicity	Intracavitary brachytherapy, improved modern stereotactic radiosurgery-targeting software
Inaccessible tumor or patient unable/unwilling to tolerate open surgery	Inability to achieve cytoreduction and tissue diagnosis	Stereotactic biopsy with MRI-guided laser interstitial thermal therapy (LITT)
Negative neurocognitive effects of whole-brain radiation	Decreased patient quality of life	Stereotactic radiosurgery and other WBRT-sparing paradigms

## Current Treatment Paradigms

Key elements driving decision-making for brain metastasis care are patient factors and tumor factors. Patient factors include the patient’s overall age, condition, and systemic disease burden, summarized as life expectancy independent of central nervous system (CNS) disease. Tumor factors include histological type, number, and location of lesions, and, more recently, the biology of the tumor based on molecular and genetic testing. Patients with poor life expectancy independent of CNS disease may reasonably be offered palliative care or no treatment for the CNS disease, regardless of the nature of the brain involvement. Conversely, patients in good medical condition with a low systemic disease burden, and hence a good survival chance independent of the brain metastases, may warrant aggressive treatment. Similarly, certain histological types of tumors (e.g., small cell lung cancer, breast cancer) are more likely to respond to adjuvant treatment with irradiation or chemotherapy, which can make their use beneficial even for numerous or poorly located lesions. Additionally, the more numerous the brain metastases, the poorer the prognosis is, irrespective of treatment. Lesions in eloquent parts of the brain (i.e., those that subserve a discrete function, such as speech or movement) or in parts of the brain less accessible *via* open neurosurgery also connote a poorer prognosis.

Neurosurgical resection of individual symptomatic brain metastases remains the standard of care. Lesions causing deficits due to local mass effect and cerebral edema should almost always undergo surgical extirpation once diagnosed, particularly if the lesion is a new diagnosis and tissue is required for pathology. Modern advances in microneurosurgical techniques and intraoperative magnetic resonance imaging-based neuronavigation allow for safe resection of lesions almost anywhere in the cerebrum. For single metastases, Patchell et al.’s landmark randomized clinical trial strongly supports surgical excision (3). Patients with a single brain metastasis underwent surgical excision followed by radiation or biopsy and radiation alone. Local control, overall survival, and quality of life were all significantly improved with surgical resection plus radiation. This study comprised mostly patients with lung cancer metastases who had high function status. Despite its lack of generalizability to all tumor patients, it remains one of the best randomized trials supporting neurosurgical intervention for brain metastases.

Traditionally, WBRT has been used after surgical resection of a single lesion or when there are multiple small asymptomatic lesions. However, WBRT carries a risk of significant cognitive morbidity, and WBRT-sparing strategies are increasingly used (4, 5). Both the American Society for Radiation Oncology and the National Comprehensive Cancer Network Clinical Practice Guidelines in Oncology have published consensus statements supporting stereotactic radiosurgery after surgical resection of a single metastasis, instead of WBRT, in patients with a single lesion and good systemic disease control (6, 7). This WBRT-sparing alternative is not supported by Level 1 randomized trial data, but rather by significant lesser strength evidence (8–12).

Lastly, depending on tumor histology and the organ of origin, standard chemotherapy is implemented at the discretion of the medical oncologist after the surgical site heals.

## Intraoperative Advances in Surgical Treatment

### Neurosurgical Resection and Tumor Visualization

Nests of tumor cells exist for several millimeters outside the confines of the distinct metastatic brain lesion and its gliotic capsule (13). Aggressive resection of this microscopic margin, when feasible, can reduce the local recurrence of brain metastases (14). Therefore, the intraoperative ability to visualize and resect these microscopic margins using fluorescence-guided surgery is of considerable interest. Fluorescence-guided neurosurgery has become commonplace in glioma surgery; various agents exploit either a degraded blood–brain barrier (e.g., fluorescein) or unique metabolism [e.g., 5-aminolevulinic acid (5-ALA)], with the goal of improving the extent of resection in infiltrative processes (15–19). Within vascular neurosurgery, indocyanine green video angiography is used in cerebral aneurysm, arteriovenous malformation, and dural arteriovenous fistula surgery as an alternative or adjuvant to traditional angiography (20–24). Multiple authors have described intraoperative fluorescence for resection of metastatic tumors, albeit with less robust support than in glioma surgery or neurovascular surgery. Schebesch and colleagues published results from a series of 30 patients with brain metastases who underwent fluorescein-guided resection using a Zeiss microscope filter system (25). Most tumors (90%) avidly expressed fluorescein, and no patients suffered complications attributable to the intravenous dye. No control group was reported, but the gross-total resection rate of 83% and permanent neurological complication rate of 6.7% are similar to reported surgical results (3, 25). Therefore, the use of fluorescein requires additional study before definitive recommendations can be made about its efficacy in improving the safe extent of resection. Some authors have noted that the area of intraoperative fluorescence seems to extend well beyond the gross tumor margins, possibly because of breakdown of the blood–brain barrier induced by the tumor. A prospective trial planned by Schebesch and colleagues will validate the usefulness of fluorescein in this setting. An alternative fluorescent agent is 5-ALA, which has found significant use in glioma surgery. Kamp et al. reported results from a retrospective series of 52 patients undergoing brain metastasis surgery using 5-ALA (26). As with fluorescein, most tumors (62%) expressed 5-ALA positivity. Residual cavity fluorescence was detected in most patients (75%) after gross-total resection of the distinct metastasis. Unfortunately, only one-third of those patients with available histological tissue samples were found to harbor microscopic disease at these 5-ALA-positive margins. Therefore, in non-eloquent areas, the use of 5-ALA seems to drive “supra-maximal” resection of surrounding reactive tissue, which means that caution is required in eloquent areas because 5-ALA positivity was not particularly sensitive for residual micrometastasis. These studies and others demonstrate the pressing need for additional research into novel fluorescent compounds to better define microscopic tumor margins in brain metastases.

### Intraoperative Diagnosis

Preoperative diagnosis of a metastatic brain tumor is not always obvious, especially in patients with no known primary malignancy and an isolated lesion. Rapid intraoperative diagnosis *via* confocal microscopy is now a viable alternative to traditional frozen sectioning with light microscopy. Given the relatively limited literature surrounding fluorescence to date, the *in vivo* application of intraoperative confocal microscopy is particularly appealing for inspection of the microscopic edges of metastatic lesions within the resection cavity itself. Our group has had success with the use of *in vivo*, real-time, handheld confocal microscopy for diagnosis of various brain tumor types and visualization of the brain–tumor interface (27–30). Further technological refinement is required for handheld confocal microscopy to be widely adopted, but it remains an appealing method to detect residual tumor.

### Brachytherapy

Radioactive brachytherapy seeds used in neurosurgery have had mixed results for a half-century (31–33). Brachytherapy enables delivery of high doses of radiation with quick dose fall-off and custom dosing to areas of residual tumor while sparing nearby radiosensitive structures *via* selective seed placement within the resection cavity. Isotypes used for intracranial brachytherapy have evolved significantly since the 1960s, with cesium-131 and iodine-125 now replacing older gold- and iridium-based therapies. Modern intracranial brachytherapy has been studied in atypical and anaplastic meningiomas, low- and high-grade gliomas, and metastases (34, 35). Most recently, the use of cesium-131 in brain metastases was reported by Wernicke et al. from a Phase I/II trial (36). Twenty-four patients underwent first-time gross-total resection of a brain metastasis and intraoperative placement of a permanent cesium-131 source with a planned dose of 80 Gy to a surface depth of 5 mm beyond the resection cavity. The patients had no local recurrences, no incidents of symptomatic radiation necrosis, and minimal surgical morbidity. This study was limited by its small size, limited follow-up, and the confounding variable of gross-total resection, which is associated with lower rates of recurrence and progression. Future studies will likely confirm these promising preliminary results with cesium-based brachytherapy for treatment of brain metastases.

### Laser Interstitial Thermal Therapy

The use of MR-guided laser interstitial thermal therapy (LITT) for metastasis has been reported in the neurosurgical literature. MR-guided thermal ablation is not a new technology, but recent advances in materials and methods have significantly improved the ability to ablate lesion tissue accurately and safely while sparing nearby brain tissue. Two series have had good results (albeit in small samples with short follow-up) for tumors that failed to respond to traditional radiotherapy and subsequently underwent LITT. The technology allows biopsy and subsequent laser ablation from a small (approximately 4 mm) access port inserted in the operating room. Carpentier et al. described four patients with six tumors treated with LITT without complications; no tumors recurred within the 90-day follow-up (37). Hawasli and colleagues demonstrated similar results using LITT for various lesions, including five metastases (38). Two patients suffered transient neurological morbidity (one aphasia and one hemiparesis), and two had progression of CNS disease 2.2 and 3.5 months after LITT. Nevertheless, LITT has good prospects as a means to extend quantity and quality of life for patients with radiation-resistant brain metastases in eloquent or deep locations who are left with few options. Furthermore, LITT can ablate radiation treatment effect found on biopsy. Further study is warranted.

## Personalized Metastasis Treatment

As our technological ability to successfully treat brain metastases has grown in recent decades, so too has our knowledge of the intricate biology of tumorigenesis. The CNS is different from other organs, as blood-borne metastatic cells must first overcome the blood–brain barrier after escaping their primary site of origin. Once these cells pass this barrier, they must establish themselves in a biological niche with a milieu of cytosolic growth factors unlike those of their site of origin. Lastly, once the cells have grown into a macroscopic tumor, different metastatic brain tumors have variable responses to irradiation and chemotherapy due to genetic and epigenetic alterations and poor penetration of the blood–brain barrier by some targeted chemotherapies. Each stage offers the potential for personalized, targeted intervention or, at least, better prognostication based on molecular (vs. histological) disease stratification. Many surgical clinical trials have grouped all metastatic tumors when evaluating new treatment strategies, but these lesions clearly have numerous biological differences despite their commonality as “brain metastases.”

### Molecular Initiation of Distal Metastases

The molecular pathophysiology of brain metastasis has been the focus of extensive research. The ability to predict, *via* primary tumor tissue, which cancer patients will suffer brain metastasis would facilitate prognostication and focus metastasis screening efforts. Numerous lung cancer researchers have attempted to correlate single gene mutations and chromosomal translocations with the development of brain metastases. For example, Lee et al. found that chromosomal amplifications of regions 5q35, 10q23, and 17q23–24 were associated with early development of brain metastases within 3 months of initial tumor diagnosis (39). The exact mechanisms by which these amplifications lead to lung-to-brain metastasis are not yet understood. Genes associated with the development of brain metastases in lung cancer include *PLGF*, *VEGFR1*, *c-MET*, and *CXCR4*, all of which are targets for further investigation (40–42). *HER2*-positivity predisposes patients with breast cancer to the development of brain metastases. This predisposition to brain metastases is probably caused by a combination of increased general *HER2*-positive tumor aggressive behavior as well as *HER2*-specific neural tropism *via* downstream pathways, such as TGF-β (43–45). Lastly, the pathophysiology of metastasis is not limited to protein-coding genes. Long non-coding RNA MALAT1 is associated with numerous cancer types and aggressive tumor behavior, despite a relatively poor understanding of the exact function of this highly preserved non-coding RNA. Regardless, high tumor levels of MALAT1 RNA are correlated with poor overall survival in patients with lung-to-brain metastasis, and MALAT1 promotes brain metastasis *via* the induction of epithelial to mesenchymal transitions (46). Further research is warranted on this long non-coding RNA, which represents an interesting non-protein target for personalized lung metastasis therapy.

### Breaching the Blood–Brain Barrier

Once individual tumor cells have hematologically spread to the cerebral microvasculature, they must exit into the perivascular space across the blood–brain barrier to propagate macroscopic tumors. Pharmacological blockade of this transgression is a highly appealing strategy to prevent the formation of brain metastases. Research on multiple tumor types has elucidated the mechanics of this process, although much work remains. An elegant murine model by Kienast and colleagues characterized the individual steps of metastasis formation as tumor cells reach the brain (47). First, individual tumor cells arrest in tiny vessel branches. Next, cells that go on to form macroscopic tumors transmigrate across the vasculature wall within 72 h after being lodged into the capillary. After transmigration, formation of a macroscopic tumor requires that tumor cells proliferate in direct contact with endothelial cells of the brain capillary akin to a pericyte. Cells that do not maintain proximity to the vessel wall regress. Lastly, vessel co-option and angiogenesis must allow for sufficient nutrient delivery to propagate the macrometastasis (47). Each step is driven by complex molecular interactions between the tumor cell and its surroundings, and all these interactions are potential targets for more directed, individualized therapies. The process of cellular transmigration out of the capillary is regulated by complex junctional adhesion molecules, and proteases that degrade these junctional adhesions are implicated in brain metastasis. For example, high levels of cathepsin S are negatively associated with overall brain-metastasis-free survival in patients with breast cancer (48). Depletion of cathepsin S in a murine model reduced *in vivo* experimental brain metastases, thus identifying another potential personalized target for those patients with high tumor cathepsin S expression (48). The degradation of the blood–brain barrier by tumor cells is regulated not only by protein-protein interactions but also by non-canonical means. Tominaga et al. demonstrated that breast cancer cells release extracellular vesicles, including microRNA such as miR-181C, which promotes the local destruction of the blood–brain barrier *via* actin fiber delocalization in a *PDPK1*-mediated fashion (49). Other exosomal microRNAs, such as miR-105, have also been implicated in the loss of cell–cell adhesion at tight junctions (50). The blockade of microRNA signaling pathways is not yet clinically practical but may represent future targeted therapies.

### Metastatic Evolution

Once established within the brain parenchyma, metastatic tumor cells continue to evolve (Table 2). Excellent genomic studies have demonstrated that brain metastases harbor gene alterations distinct from the primary tumor. These alterations have widespread ramifications, especially for patients with inoperative brain metastases whose primary lesion is the only tissue available for molecular profiling for additional therapy selection. Brastianos et al. found that in 53% of tumors, clinically relevant alterations occurred in the brain metastasis but not in the primary tumor (51). Many of these mutations arose in the *PI3K*/*AKT*/*mTOR*, *CDK*, and *HER2*/*EGFR* pathways, all of which have inhibitors available for clinical use. In this same cohort, multiple distinct brain lesions were genetically homogeneous compared to the extracranial metastases (51). This genetic homogeneity has significant practical implications because personalized targeted therapies for multiple brain lesions are best chosen on the basis of molecular data from any single brain metastasis rather than from the more divergent primary tumor or extracranial metastatic disease. Similar results demonstrate significant genetic divergence between brain metastasis and primary tumor tissue, specifically for squamous cell lung cancer (52). Further DNA- and RNA-based high-throughput sequencing comparing primary tissue and brain metastases will shed additional light on the metastatic process (and subsequent potential therapies) in coming years.

**Table 2:** **Examples of metastatic events, their molecular processes, and potential targeted chemotherapeutics**.

Cellular event	Pathway(s) implicated	Potential personalized treatments
Migration across BBB	cathepsin S	Inhibitors in development
	miR-181C	None to date
	miR-105	None to date
Survival in CNS microenvironment	mTOR	Everolimus, temsirolimus
	CDK	Palbociclib, others in development
	VEGF	Bevacizumab
	EGFR	Erlotinib
Establishment of radioresistance	Chk1	Inhibitors in development
	c-Met	Cabozantinib

### Targeted Drug Delivery

Personalization of metastatic cancer treatment aims to improve treatment by using select therapies chosen *via* molecular profiling to benefit the patient while sparing the patient from biologically irrelevant therapies with potential toxicity. In brain metastases, this strategy is limited because of poor penetration of most novel chemotherapeutics across the blood–brain barrier. Earlier research has used mannitol as a non-specific agent for blood–brain barrier permeation with limited success (53). Researchers have since demonstrated the ability to permeate the blood–brain barrier selectively in murine models at the site of metastases using intravenous tumor necrosis factor (54). Although in its infancy, MR-focused ultrasound combined with microbubbles represents another method of blood–brain barrier disruption but requires significant dedicated infrastructure (55). Many novel targeted delivery strategies are in development in multiple centers, making it likely that more options will become available in the future.

### Advances in Radiotherapy

At times, the molecular biology of radiation resistance can be overcome, allowing for more effective delivery of radiotherapy. Xenograft brain metastasis in murine models have demonstrated improved survival and response to external beam radiation after inhibition of Chk1 (DNA damage checkpoint protein) and c-Met (receptor tyrosine kinase with downstream oncogenes) (56–59). Clinical trials are required to demonstrate a benefit in human patients.

## Summary and Future Directions

Brain metastases represent a common source of morbidity and mortality for cancer patients. Current treatment paradigms include surgical resection, radiotherapy, and chemotherapy. Recent advances in intraoperative surgical technology (i.e., fluorescence, confocal microscopy, and brachytherapy) hold promise for improved outcomes for brain metastasis resection. The future of brain metastasis management is predicated on personalized therapy targeted to specific tumor molecular pathways, such as those involved in blood–brain barrier transgression, cell–cell adhesion, and angiogenesis. Brain metastases are often biologically distinct lesions compared to the primary tumor. Personalized therapies should therefore be chosen on the basis of brain metastasis tissue whenever available. The multidisciplinary management of patients with brain metastases by neurosurgeons, medical oncologists, and radiation oncologists is essential as therapies become increasingly complex and individualized.

## Author Contributions

All authors listed have made substantial, direct, and intellectual contribution to the work and approved it for publication.

## Conflict of Interest Statement

The authors declare that the research was conducted in the absence of any commercial or financial relationships that could be construed as a potential conflict of interest.

## Abbreviations

5-ALA, 5-aminolevulinic acid; CNS, central nervous system; LITT, laser interstitial thermal therapy; WBRT, whole-brain radiotherapy.
